# Immune regulation of ovarian development: programming by neonatal immune challenge

**DOI:** 10.3389/fnins.2013.00100

**Published:** 2013-06-12

**Authors:** Luba Sominsky, Alexander P. Sobinoff, Matthew S. Jobling, Victoria Pye, Eileen A. McLaughlin, Deborah M. Hodgson

**Affiliations:** ^1^Laboratory of Neuroimmunology, Faculty of Science and IT, School of Psychology, The University of NewcastleCallaghan, NSW, Australia; ^2^PRC in Reproductive Science and Chemical Biology, Faculty of Science and IT, School of Environmental and Life Sciences, The University of NewcastleCallaghan, NSW, Australia

**Keywords:** LPS, TLR4, MAPK, neonatal, immune challenge, reproductive development

## Abstract

Neonatal immune challenge by administration of lipopolysaccharide (LPS) produces enduring alterations in the development and activity of neuroendocrine, immune and other physiological systems. We have recently reported that neonatal exposure to an immune challenge by administration of LPS results in altered reproductive development in the female Wistar rat. Specifically, LPS-treated animals exhibited diminished ovarian reserve and altered reproductive lifespan. In the current study, we examined the cellular mechanisms that lead to the previously documented impaired ovulation and reduced follicular pool. Rats were administered intraperitoneally either 0.05 mg/kg of LPS (*Salmonella Enteritidis*) or an equivalent volume of non-pyrogenic saline on postnatal days (PNDs) 3 and 5, and ovaries were obtained on PND 7. Microarray analysis revealed a significant upregulation in transcript expression (2-fold change; *p* < 0.05) for a substantial number of genes in the ovaries of LPS-treated animals, implicated in immune cell signaling, inflammatory responses, reproductive system development and disease. Several canonical pathways involved in immune recognition were affected by LPS treatment, such as nuclear factor-κB (NF-κB) activation and LPS-stimulated mitogen-activated protein kinase (MAPK) signaling. Quantitative Real-time PCR analysis supported the microarray results. Protein expression analysis of several components of the MAPK signaling pathway revealed a significant upregulation in the expression of Toll-like receptor 4 (TLR4) in the neonatal ovary of LPS-treated animals. These results indicate that neonatal immune challenge by administration of LPS has a direct effect on the ovary during the sensitive period of follicular formation. Given the pivotal role of inflammatory processes in the regulation of reproductive health, our findings suggest that early life immune activation via TLR signaling may have significant implications for the programming of ovarian development and fertility.

## Introduction

Worldwide there is a trend for declining fertility. This is particularly apparent for the female population, whereby 10.9% of women suffer from impaired fertility and 6% are infertile (Martinez et al., 2012). Decreased female fecundity has been related to advanced maternal age but the increasing representation of young women in this category (Martinez et al., 2012), suggests a more complex epidemiology. Accumulating evidence points to the critical role of the early life environment in the establishment of reproductive health. Substantial epidemiological and experimental evidence indicates that adult physiological function and health status may have their origins in the early developmental period (Barker and Osmond, 1986; Barker et al., 1989; Barker, 1990). Plasticity during the perinatal period allows adequate adaptation of an organism to given environmental conditions and predicts later life functioning. While physiological plasticity holds a beneficial value for the developing organism, exposure to adverse environmental conditions during critical maturational periods, may result in alterations to the normal developmental trajectory, increasing susceptibility to physiological malfunction and pathology in later life. The process by which early life environment can have permanent effect on physiological systems has been described as *perinatal programming* (Welberg and Seckl, 2001; Davies and Norman, 2002).

One of the major physiological systems that undergoes development and maturation during foetal and early postnatal life is the immune system. Due to functional immaturity of the neonatal immune system in both animals and humans, there is increased susceptibility to infections and a lower response to immunogenic stimuli when compared to that of the adult (Vosters et al., 2010). The impaired immune responses, upon exposure to infectious agents, during the perinatal period have been associated with the decreased capacity of the neonate to develop mature protective T helper type 1 immune responses (De Wit et al., 2003). As such, low levels of proinflammatory cytokines have been detected in response to immune stimulation by LPS and *Staphylococcus epidermidis* as documented in neonatal rats and mice, as well in human cord blood (Angelone et al., 2006; Hartel et al., 2008; Hodyl et al., 2008). The development of the immune system is well-established to be dependent on the immune, autonomic and endocrine signals that it receives early in life (Holladay and Smialowicz, 2000; Zakharova, 2009; Fagundes et al., 2012). This physiological programming of the immune system and its relationship to later life pathology has been associated with a number of long-term health outcomes mediated by inflammatory pathways including, a predisposition to asthma, allergies, autoimmune diseases, metabolic disorders, cardiovascular diseases, multiple sclerosis, and more (Zakharova, 2009; Fagundes et al., 2012), depending on the timing and the extent of early life immunogenic exposure.

Programming of the immune system has been investigated using various animal models. Experimentally-induced early life immune activation is commonly achieved by administration of lipopolysaccharide (LPS) [derived from *Salmonella Enteritidis* or *Escherichia (E.) coli*], the cell wall of Gram-negative bacteria, in rodents. LPS is recognized by the Toll-like receptor 4 (TLR4) that is expressed by several cell types, such as monocytes, macrophages, adipocytes as well as gonadal supporting cells (i.e., ovarian granulosa and testicular sertoli cells) (Medzhitov, 2001; Richards et al., 2008). The impact of neonatal LPS exposure has been examined on a variety of physiological and behavioral outcomes, such as adult immune responses (Boisse et al., 2004; Spencer et al., 2006; Walker et al., 2009b; Mouihate et al., 2010), metabolism (Walker et al., 2006; Iwasa et al., 2009a), brain morphology (Bilbo et al., 2008; Amath et al., 2012; Sominsky et al., 2012b), stress responsivity, and anxiety-like behaviors (Walker et al., 2009a, 2012; Sominsky et al., 2013).

The reciprocal relationship between the hypothalamic-pituitary-gonadal (HPG) axis and the immune system has an extensive impact on development and functioning of both systems, as well as on health outcomes. Oestrogen and androgen receptors are expressed on immune cells (Tanriverdi et al., 2003), and these in turn have been shown to populate all reproductive organs (Seamark et al., 1992). Recently, increasing attention has focused on the effect of neonatal immune challenge on reproductive development and functioning. Activation of the immune system during neonatal life has long-term consequences for reproductive functioning (Marchetti et al., 2000; Morale et al., 2003). Experimental evidence has indicated altered puberty onset, diminished HPG axis activity and impaired sexual behavior in both male and female rodents neonatally exposed to bacterial endotoxin, LPS, in particular on PNDs 3 and 5 (Iwasa et al., 2009b,c; Knox et al., 2009; Walker et al., 2011; Wu et al., 2011; Sominsky et al., 2012a), suggesting that these changes may be attributed to the critical window of reproductive development. Supporting this assertion, the critical period for programming of pubertal onset in the female rat has been determined to be before 7 days of age. LPS treatment on PNDs 3 and 5 has been shown to result in a significant delay in puberty, while LPS administered on PNDs 7 and 9 or PNDs 14 and 16 produced no such effect (Knox et al., 2009). Furthermore, neonatal LPS exposure has been demonstrated to aggravate LPS-induced suppression of LH pulse frequency in adulthood, when compared to neonatal saline-treated group (Li et al., 2007). Similarly, neonatal LPS-treated female rats exhibited prolonged oestrous cycle in response to LPS injection in adulthood, while no such response was observed in neonatal saline-treated animals (Iwasa et al., 2009c).

Neonatal LPS exposure has also been reported to produce alterations in gonadal morphology. Reduced gonocyte populations in neonatal testes, as well as increased epithelial disorganization and delayed spermatogenesis in adulthood were demonstrated in male rats neonatally exposed to LPS (Walker et al., 2011). Robust alterations in gonadal morphology in response to neonatal LPS challenge have also been documented in female animals. Diminished follicular reserve has been detected in the ovaries of LPS-treated female rats (Wu et al., 2011), with reduced population of primordial follicles being evident at 2 weeks of age, after LPS exposure on PNDs 3 and 5 (Sominsky et al., 2012a). This later finding corresponded with advanced senescence and poor pregnancy outcomes in LPS-treated females (Sominsky et al., 2012a), suggesting a prolonged and more robust effect of an immune challenge on female reproductive lifespan. Of particular relevance to the model of neonatal immune challenge, is that the initial follicular assembly and maturation are known to be dependent on local inflammatory agents, such as growth factors and cytokines (Dissen et al., 2002; Skinner, 2005; Schindler et al., 2010). Even after puberty, when ovarian function is largely governed by the HPG hormones (McGee and Hsueh, 2000), ovulation resembles an inflammatory process, as constituted by increased vasodilation and hyperaemia of ovarian follicles, and their ability to produce cytokines, chemokines, and prostaglandins (Espey, 1980; Richards et al., 2002). This concept is further strengthened by the abundance of macrophages, principal innate immune cells, in the ovary, contributing to the regulation of ovarian function (Wu et al., 2004). Furthermore, ovarian granulosa and cumulus cells exert several immune-related functions, including the expression of TLR4, which during ovulation respond to endogenous ligands, resulting in the release of pro-inflammatory cytokines, such as IL-6 (Richards et al., 2008).

While immune-regulation of ovarian function is crucial for normal fertility, any alteration in this delicate relationship may lead to ovarian dysfunction and subfertility. Common ovarian diseases, such as polycystic ovarian syndrome (PCOS) and endometriosis, have been associated with increased levels of follicular and serum pro-inflammatory cytokines, constitutive of chronic low grade inflammation (Wu et al., 2004). The direct impact of LPS exposure on ovarian function has been previously assessed *in-vitro*, and was reported to result in diminished follicular function (Herath et al., 2007) and impaired oocyte meiotic competence (Bromfield and Sheldon, 2011). No studies have investigated *in vivo* the mechanisms that underpin the effect of peripheral LPS exposure on ovarian development during the neonatal period. In the current study, we aimed to identify the ovarian pathways that lead to the previously documented impaired ovulation and reduced oocyte development, in the model of dual LPS exposure on PNDs 3 and 5 (Knox et al., 2009; Wu et al., 2011; Sominsky et al., 2012a). In order to characterize the cellular mechanisms that underpin the immediate effect of peripheral LPS exposure on PNDs 3 and 5 on ovarian development, we examined its effect on the ovarian transcriptome on PND 7. Given that in the rat, the assembly of primordial follicles is not established until PND 3 (Rajah et al., 1992; Skinner, 2005), we propose that an immune challenge by administration of LPS at this time point may directly intervene with the formation and establishment of the finite follicular pool via activation of inflammatory pathways.

## Methods

### Animals and neonatal treatment

All animal experimental procedures were conducted with the approval of the University of Newcastle Animal Care and Ethics Committee (ACEC). Seven experimentally naive female Wistar rats were obtained from the University of Newcastle animal house and mated in the University of Newcastle Psychology vivarium. Animals were maintained under normal housing conditions at 21–22°C, under a 12 h light/dark regime. At birth (PND 1), whole litters were randomly allocated into either LPS (4 litters; litter size *M* = 13, *SD* = 1.8) or saline-treated conditions (3 litters; litter size *M* = 15, *SD* = 0). As documented previously (Walker et al., 2004, 2006, 2009a,b, 2010; Sominsky et al., 2012a,b), on PNDs 3 and 5 pups were briefly removed from their home cages, weighed and administered intraperitoneally with either LPS (Salmonella enterica, serotype enteritidis; Sigma-Aldrich Chemical Co., USA, dissolved in sterile pyrogen-free saline, 0.05 mg/kg) or an equivolume of saline (Livingstone International, Australia). No significant differences in neonatal weight were observed when assessed on PND 3, PND 5, and PND 7 in the female offspring. We have previously reported neonatal LPS treatment to produce variable effects in regards to the neonatal weight gain, inducing weight loss (Walker et al., 2004; Sominsky et al., 2012a), weight gain (Walker et al., 2011) or no significant change (Walker et al., 2009a). Litter size and male-to-female ratio were not significant covariates to this analysis, confirming that these factors did not contribute to the possible developmental effects of litter.

On PND 7, female pups were euthanized by rapid decapitation. Ovaries were collected, frozen on dry ice and stored at −80°C until further analysis. For microarray and qRT-PCR analyses, ovaries were obtained from 5 animals per treatment group (derived from 2 LPS and 2 saline-treated litters). For western blotting analysis, ovaries were obtained from 4 animals per treatment group (derived from 2 LPS and 1 saline-treated litters). An additional subset of ovarian samples was used for the exploratory immunohistochemical analysis. Ovaries were obtained from 3 LPS and 3 saline-treated animals (derived from 2 LPS and 2 saline-treated litters) on PND14. This later time point was chosen to ensure a complete structural representation of the neonatal ovary, as at this age the ovary has been shown to contain antral follicles, which are completely absent prior to 12 days of age (Carson and Smith, 1986).

### RNA extraction

RNA was isolated from ovaries using RNeasy Mini Kit (Qiagen Inc., Valencia, CA, USA) in accordance with the manufacturer's instructions, with slight modifications. Briefly, ovaries were homogenized in lysis buffer (RNeasy buffer RLT) using a basic plastic homogenizer. The homogenized RNA was then passed five times through a 20 gauge needle. Lysate was centrifuged at 13,000 rpm for 3 min at 25°C. The supernatant was collected, 350 μl 70% ETOH added and mixed. The solution was then transferred to an RNeasy spin column and placed in a 2 ml collection tube. Following 20 s centrifugation at 10,000 rpm the flow through was discarded. Seven hundred micro liters of wash buffer (RNeasy buffer RW1) was added to the RNeasy spin column, centrifuged for 20 s at 10,000 rpm, following which the flow through was discarded. This step was repeated twice with 500 μl of RNeasy buffer RPE. RNeasy spin column was placed in a new 1.5 ml collection tube, add 30 μl RNase-free H_2_O were added directly to the column membrane and centrifuged for 1 min at 10,000 rpm to collect RNA. RNA concentrations were determined using spectrophotometer, NanoDrop 2000c (Thermo Fisher Scientific, Wilmington, DE USA) (refer to Table S1 for RNA quality information). The extracted RNA was used for microarray analysis and for verification by qRT-PCR.

### Microarray analysis

Total RNA obtained from neonatal ovaries (1–2 μg) was submitted to the Australian Genome Research Facility (AGRF). Microarray analysis was performed using Agilent SurePrint G3 Rat GE 8x60K Array platform. Total RNA obtained from neonatal ovaries was quality ascertained on the Agilent Bioanalyser 2100 using the NanoChip protocol (Table S1). A total of 1 μg was labeled using the Ambion Total Prep RNA amplification kit (Applied Biosystems—AMIL1791). The quantity of amplified product was ascertained using the Agilent Bioanalyser 2100 using the NanoChip protocol. For the Agilent 8x60k chip format, 600 ng of amplified cRNA was used for the Cy3 coupling process using a ULS labeling kit (Kreatech EA-006). After coupling the dye, the reaction was cleaned using the KREApure columns in the kit. The Cy3 labeled cRNA samples were then fragmented using the Ambion fragmentation kit (Applied Biosystems—AM8740). The fragmented cRNA was quality checked on the Agilent Bioanalyser 2100 using the NanoChip protocol. The fragmented Cy3 labeled cRNA was then prepared for hybridization to the Agilent 8x60k Rat array using the GX hybridization kit (Agilent—5188-5242) where each hybridization reaction had a final volume of 50 μl. The Agilent hybridization chambers were prepared according to manufacturer's instructions. Each sample was loaded onto the Agilent hybridization gaskets slide which is placed into a hybridization chamber. The 8x60k Rat array was carefully lowered onto the gasket to create a sealed array for each sample. The hybridization chambers were then placed in a rotating hybridization oven at 65°C for 17 h. After hybridization, the chip was washed using the appropriate protocols as outlined in the Agilent manual using the GX washing buffers (Agilent—5188-5327). Upon completion of the washing, the chips were then scanned in the Agilent DNA Microarray Scanner (High Resolution). The scanner operating software, Agilent Scan Control, converts the signal on the array into a TIFF file which can be used for subsequent analysis. The experiment was conducted in three biological replicates, which consisted of ovarian tissue obtained from five animals per treatment group. Differentially expressed genes were determined by a twofold difference and a significance level of *p* < 0.05. Ingenuity Pathways Analysis (Ingenuity Systems, Redwood City, CA) software was used for further analysis to identify canonical signaling pathways affected by LPS treatment. The data discussed in this publication have been deposited in NCBI's Gene Expression Omnibus and are accessible through GEO Series accession number GSE46436 (http://www.ncbi.nlm.nih.gov/geo/query/acc.cgi?acc=GSE46436).

### Reverse transcription and quantitative real-time PCR

Reverse transcription was performed with 1 μg of total RNA using a SuperScript® VILO™ cDNA Synthesis Kit (Invitrogen Corp., Carlsbad, CA, USA), according to manufacturers' instructions, by combining the components [5× VILO reaction mix (4 μ L), 10× SuperScript enzyme mix (2 μ L), RNA sample (up to 1 μg), diethyl pyrocarbonate (DEPEC) treated water (to 20 μ l)]. Tube contents were gently mixed, incubated at 25°C for 10 min, and then incubated at 42°C for 60 min. The reaction was terminated at 85°C at 5 min. qRT-PCR was performed using SYBR Green PCR Master Mix (Invitrogen, Carlsbad, CA, USA) on a 7500 RT-PCR Fast instrument (Applied Biosystems, Foster City, CA, USA). Primer sequences were designed using the Primer3 software (http://frodo.wi.mit.edu). Sequence specificity was tested using the Basic Local Alignment Search Tool at NCBI (Altschul et al., 1997) and primer pairs were obtained from Invitrogen (custom DNA oligonucleotide synthesis service). PCR efficiency for each pair of primers was determined by a standard curve method, where *C*(*t*) values for serial dilutions are related to the logarithm of the dilution factor, and the slope is a measure for reaction efficiency. Information regarding primer sequences, including product size and PCR efficiency, is listed in Table S2. The 25 μl PCR mixture consisted of 12.5 μl SYBR Green PCR Master Mix, 9.5 μl water and 2 μl of each primer was added to 1 μl of the cDNA template (10 ng/ml). All reactions were performed in duplicate under the following conditions: 95°C for 20 s and 40 cycles of 95°C for 3 s and 60°C for 30 s. Melting curve was determined under the following conditions: 95°C for 15 s, 60°C for 1 min, 95°C for 15 s and 60°C for 15 s. The data were normalized to an endogenous control, β-actin. A relative quantitative measure of the target gene expression compared with β-actin mRNA was analysed using the equation 2^−ΔΔ*C*(*t*)^, where *C*(*t*) is the threshold cycle at which fluorescence is first detected as statistically significant above background, and presented as a fold increase relative to the saline control.

### Protein extraction and western blotting

Protein was extracted using RIPA buffer (150 mM NaCl, 0.5% sodium deoxycholate, 0.1% SDS, 50 mM Tris, pH 8, 1% Triton X-100), supplemented with a protease inhibitor cocktail (ProteCEASE, G-Biosciences St. Louis, MO, USA). Protein concentration was determined using a Pierce BCA Protein Assay Kit (Thermo Scientific, Rockford, IL, USA). Quantified aliquots were separated by electrophoresis and transferred onto a nitrocellulose Hybond C-Extra membrane (Amersham) prior to blocking for 1 h in 5% skim milk powder in TBST (0.1% Tween-20), as previously described (Sobinoff et al., 2010). After blocking, the membranes were incubated overnight at 4°C with goat polyclonal TLR4 (L-14) (diluted 1:200; Santa Cruz, sc-16240); goat polyclonal PKCβ1 (C-16) (diluted 1:500; Santa Cruz, sc-209); or rabbit polyclonal anti-JNK1 (diluted 1:2000; Abcam, ab10664) primary antibodies. Following washing and incubation with horseradish peroxidase-conjugated donkey anti-goat secondary antibody (Santa Cruz, sc-2020) at a 1:3000 dilution or goat anti-rabbit secondary antibody (Millipore DC03L) at a 1:2000 dilution for 1 h at room temperature, proteins were visualized using an ECL Detection Kit (GE Healthcare Life Sciences) according to manufacturer's instructions. The membranes were then stripped of primary and secondary antibodies in Western Re-Probe (G-Biosciences) according to the manufacturer's instructions at room temperature for 1 h and reprobed using a mouse monoclonal anti-α-tubulin (Sigma, T5168) as a loading control. The densities of TLR4, PKCβ1 and JNK1 bands were visualized using ImageQuant LAS 4000 imager (GE Healthcare Life Sciences). The protein bands were measured using MultiGauge V3.0 (Fuji, Stamford, CT, USA) software and were expressed as the ratio to α-tubulin.

### Immunohistochemistry

Immunohistochemistry was used to localize TLR4, PKCβ1, and JNK1 proteins in the neonatal ovaries and for proliferating cell nuclear antigen (PCNA) detection of primordial follicle activation and growth. Ovaries were obtained from LPS and saline-treated animals on PND14. Tissue was fixed in Bouin's fixative solution for 4 h, washed four times in 70% ethanol dehydrated, embedded in paraffin and sectioned at 4 μm. Slides were dewaxed in xylene and rehydrated with subsequent washes in ethanol. Antigen retrieval was carried out by microwaving sections for 3 × 3 min in Na Citrate buffer (10 mM Sodium Citrate, pH 6) for TLR4, PKCβ1, and PCNA, and in Tris buffer (50 mM, pH 10.6) for JNK1. Slides were allowed to cool before being blocked in 3% BSA/Tris-buffered saline (TBS) for 1 h at room temperature. Sections were incubated overnight at 4°C with anti-TLR4 (diluted 1:100; Santa Cruz, sc-16240); anti-PKCβ1 (1:200, Santa Cruz, sc-209) and mouse monoclonal anti-PCNA (diluted 1:200; Merck KGaA, NA03T). For detection of anti-JNK1 (diluted 1:500, Abcam, ab10664) sections were incubated for 2 h at room temperature. The above primary antibodies were used for Western blotting prior to their application for the immunohistochemical analysis, ascertaining antibodies specificity (Kurien et al., 2011). Slides were then washed in TBS containing 0.1% Triton X-100, and incubated with the appropriate fluorescent-conjugated secondary antibodies (Alexa Fluor 594 donkey anti-goat IgG; Alexa Fluor 594 goat anti-rabbit IgG and Alexa Fluor 594 goat anti-mouse IgG; Invitrogen; 1:200 dilution). Sections were then counterstained with 4#-6-diamidino-2-phenylindole (DAPI) for 2 min, mounted in Mowiol (4-88, Sigma), viewed using an Axio Imager A1 fluorescent microscope (Carl Zeiss MicroImaging, Inc., Thornwood, NY) under fluorescent optics and pictures taken using an Olympus DP70 microscope camera (Olympus America, Center Valley, PA).

### Terminal deoxynucleotidyl transferase dUTP nick end labeling (TUNEL) analysis

Bouin's fixed sections were dewaxed and rehydrated as mentioned above. Slides were then boiled in Tris buffer (50 mM, pH 10.6) for 20 min and treated with 20 μg/ml Proteinase K for 15 min in a humidified chamber. TUNEL analysis was then performed as previously described (Sobinoff et al., 2010, 2011, 2012) using an *In Situ* Cell Death Detection Kit, Fluorescein (Roche Diagnostics Pty Ltd), according to the manufacturer's instructions. Slides were then counterstained with DAPI for 5 min, mounted in Mowiol, and viewed using an Axio Imager A1 fluorescent microscope (Carl Zeiss) under fluorescent optics and pictures taken using an Olympus DP70 microscope camera (Olympus).

### Data analysis

Statistical analyses were conducted using the Statistical Package for the Social Sciences for Windows, Version 18 (SPSS Inc.). Data were analysed using analyses of variances (ANOVA) design. The significance level was set at *p* ≤ 0.05.

## Results

### Impact of LPS treatment on the neonatal ovarian transcriptome

**Figure 1:**
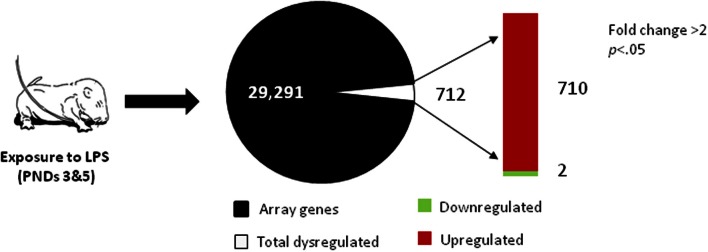
**Microarray analysis of neonatal ovaries obtained from LPS-treated vs. saline-treated animals, on PND 7**. RNA was isolated and subjected to microarray analysis as described in the Methods section. Total number of genes present on an Agilent SurePrint G3 Rat GE 8x60K Array are presented as non-regulated (black) and regulated (white) genes with a significant change in expression (≥2-fold change, *p* < 0.05). The red bar represents the number of upregulated genes and the green bar represents the number of downregulated genes in the ovaries of LPS-treated animals.

Administration of LPS on PNDs 3 and 5 caused a significant change in ovarian gene expression for 712 genes, representing 2.4% of the total number of genes present on the array, with significant upregulation of 710 genes (Figure 1; Table S3). Further functional analysis using Ingenuity Pathway Analysis software identified these significantly altered genes as components of several molecular networks implicated in cell signaling, immune cell trafficking, inflammatory response as well as reproductive development and disease (see Table 1). These results suggest a robust impact of peripheral immune challenge on a variety of molecular mechanisms regulating ovarian development and function. No distinct function has been assigned to the two significantly downregulated genes, *SBK2* and *TEDDM1*.

**Table 1:** **Functional categorization of genes that were upregulated by neonatal LPS exposure**.

**Molecular and cellular function**	**Upregulated genes**
Inflammatory response	50
Immune cell trafficking	29
Inflammatory disease	54
Organismal development	38
Developmental disorder	39
Reproductive system development and function	29
Reproductive system disease	28
DNA replication, recombination, and repair	34
Cell morphology	32

Significantly upregulated genes were analysed using Ingenuity Pathway Analysis software for molecular and cellular functions. Only those genes exhibiting a greater than two-fold change in expression were analysed (p < 0.05). Some genes are listed in multiple functional groups.

### Canonical pathways significantly upregulated by neonatal LPS exposure

**Figure 2:**
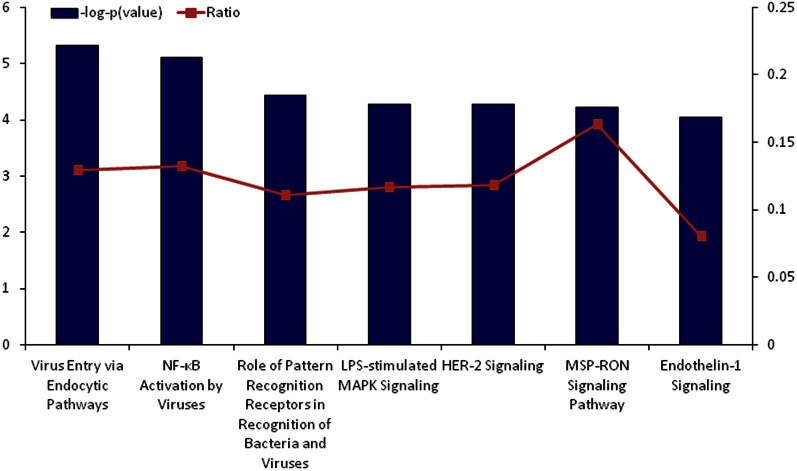
**Top canonical pathways that were significantly upregulated by neonatal LPS treatment, as identified by Ingenuity Pathway Analysis**. The significance of association between upregulated genes and the canonical pathway was assessed using a right-tailed Fisher's exact test to calculate a *p*-value determining the probability that the association is explained by chance alone (blue bars, y-axis). Ratios defining the proportion of upregulating genes from a pathway related to the total number of molecules that make up that particular pathway are also displayed (line graph, z-axis).

**Figure A1:**
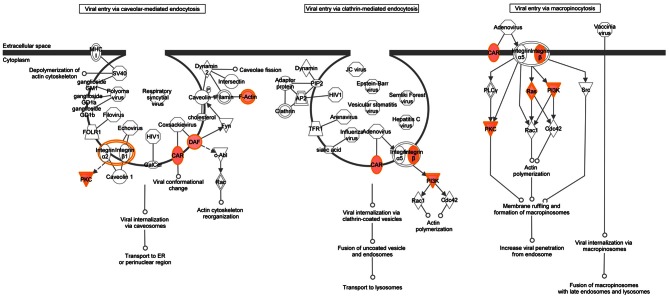
**Virus entry via endocytic pathways**.

**Figure A2:**
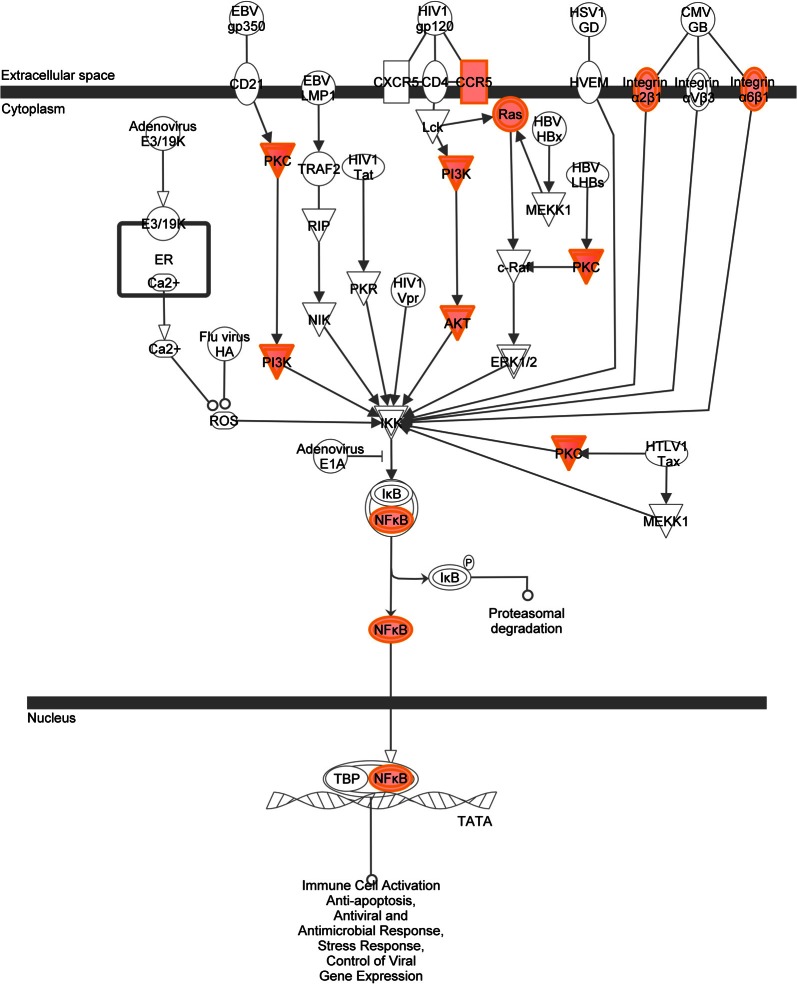
**NF-κB activation by viruses**.

**Figure A3:**
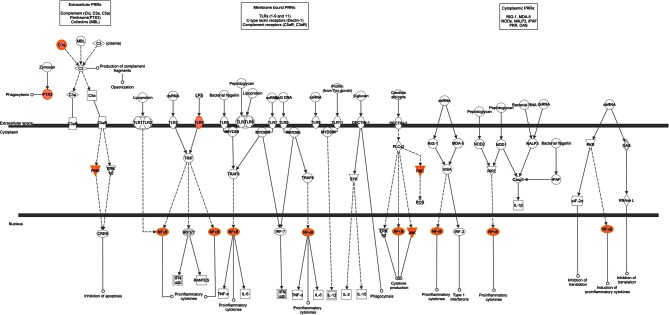
**Role of pattern-recognition receptors in recognition of bacteria and viruses**.

**Figure A4:**
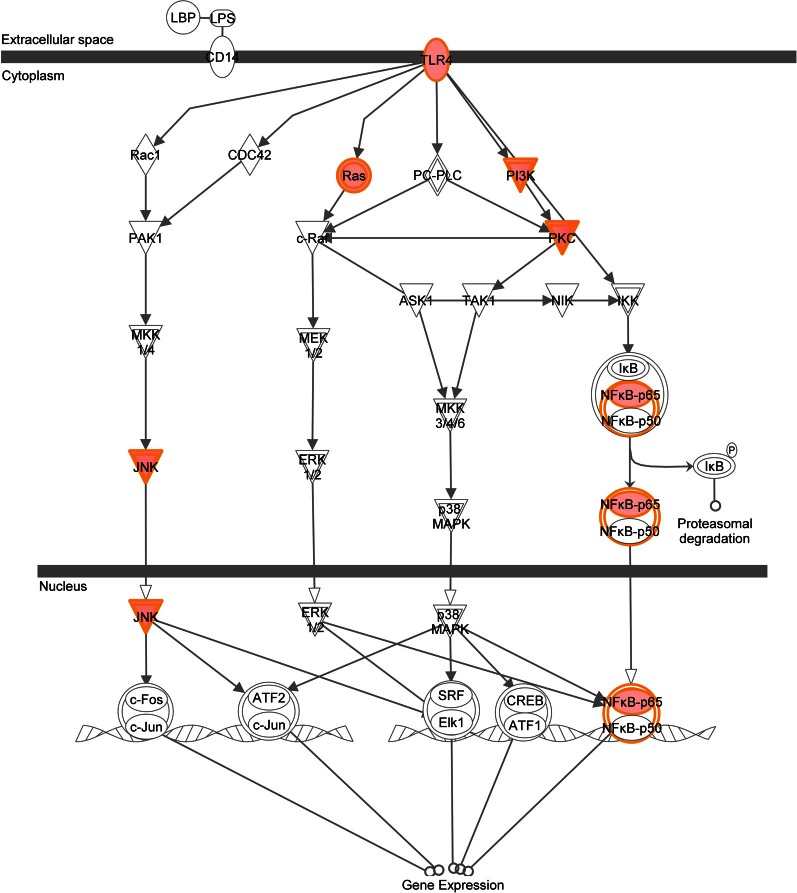
**LPS-stimulated MAPK signaling**.

**Figure A5:**
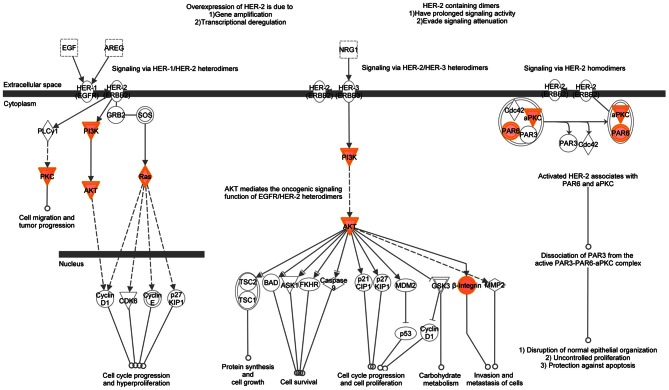
**HER-2 signaling in breast cancer**.

**Figure A6:**
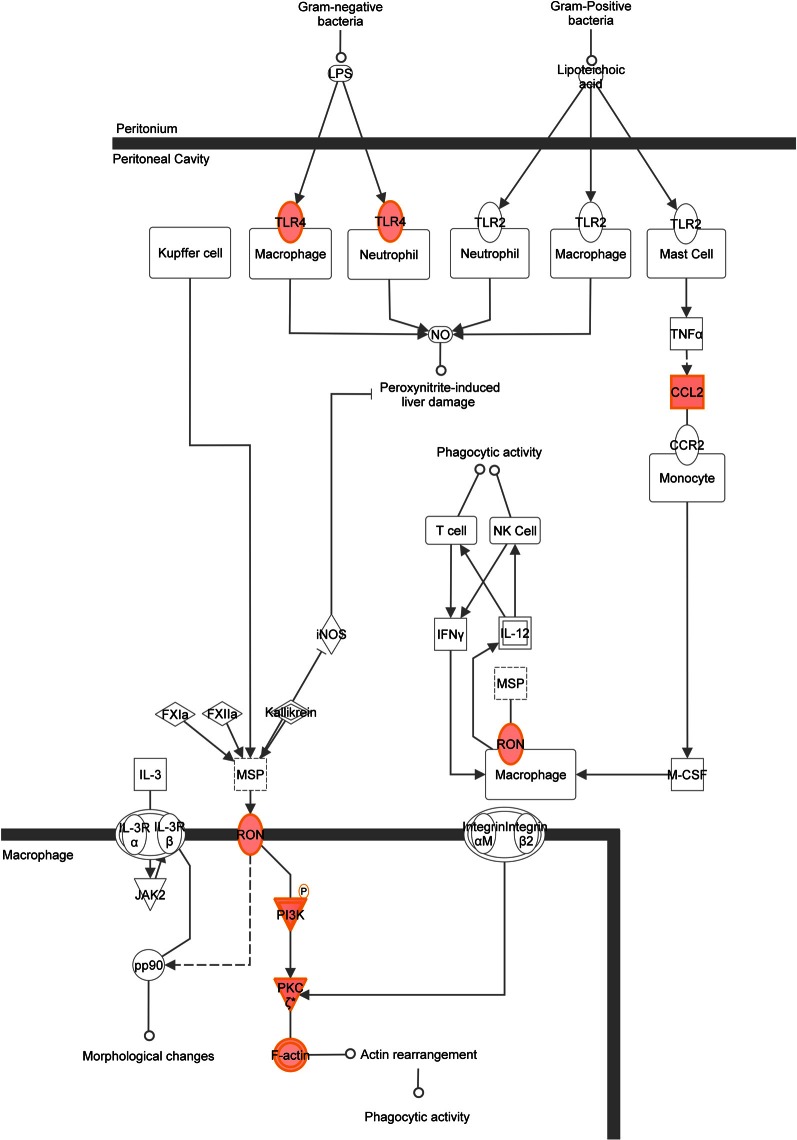
**MSP-RON signaling pathway**.

**Figure A7:**
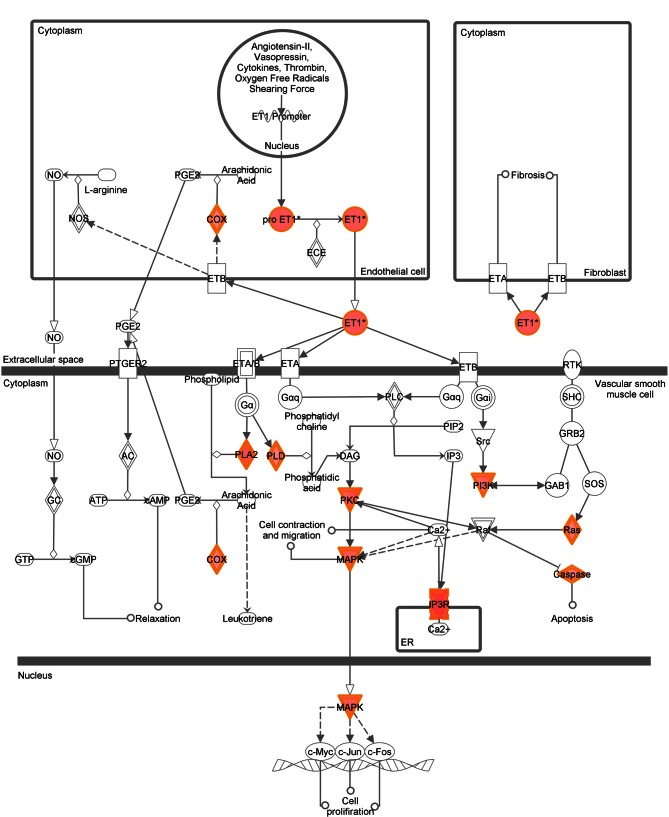
**Endothelin-1 signaling**.

Further functional assessment of differentially regulated genes revealed several canonical pathways involved primarily in immune recognition and inflammation were activated in response to neonatal treatment [e.g., virus entry via endocytic pathways; nuclear factor-kappaB (NF-κB) activation; mitogen-activated protein kinase (MAPK) signaling; pattern-recognition receptors signaling; MSP-RON signaling, whereby macrophage-stimulating protein (MSP), acts through the RON receptor tyrosine kinase, and plays a regulatory role in the inflammatory response], suggesting dysregulation of inflammatory processes in response to peripheral LPS exposure may occur also locally, in the ovary (Figure 2; Table S4). Figures A1–A7 provide a graphical representation of the significantly altered genes in the top canonical pathways.

### qRT-PCR confirmation of microarray analysis

LPS-stimulated MAPK signaling pathway was chosen for validation of microarray results, due to its specific relevance to the current model of LPS challenge. qRT-PCR analysis confirmed significant upregulation of 8 out of 9 genes that were identified as components of this pathway, in the ovaries of LPS-treated animals (Table 2). Of these genes, upregulation of *MAPK8/JNK1* has been previously associated with oocyte dysfunction (Sobinoff et al., 2010); *PKC*β has been reported to be involved in oocyte activation (Carbone and Tatone, 2009); *PIK3C2A* has been implicated in proliferation of ovarian theca-interstitial cells (Kwintkiewicz et al., 2006); *PIK3R1* and *PKCZ* have been associated with the incidence of PCOS (Diamanti-Kandarakis, 2008; Kim et al., 2009; Rivero et al., 2012); *PKD3* has been implicated in organogenesis (Ellwanger et al., 2008); *RELA* and *RRAS2* have been associated with ovarian tumorigenesis (Chan et al., 1994; Niesporek et al., 2007; Fan and Richards, 2010); and finally, increased expression of *TLR4* (receptor for LPS) has been demonstrated to underpin impaired follicular growth and function (Herath et al., 2007).

**Table 2:** **qRT-PCR confirmation of microarray results for the components of LPS-stimulated MAPK signaling pathway, upregulated in the ovaries of LPS-treated animals**.

**Gene symbol**	**Gene name**	**Summary of function**	**Fold change**
*MAPK8/JNK1*	Mitogen-activated protein kinase/c-Jun N-Terminal Protein Kinase 1	Increased expression is associated with xenobiotic-induced oocyte dysfunction (Sobinoff et al., 2010)	2.99 ± 0.04^*^
*PIK3C2A*	Phosphatidylinositol-4-phosphate 3-kinase, catalytic subunit 2A	Involved in modulation of proliferation of ovarian mesenchyme (Kwintkiewicz et al., 2006)	2.74 ± 0.04^*^
*PIK3R1*	Phosphoinositide-3-kinase, regulatory subunit 1	Involved in the insulin receptor signaling pathway; associated in the pathogenesis of PCOS (Kim et al., 2009)	3.32 + 0.04^*^
*PKC*β	Protein kinase C, beta	Involved in oocyte activation. Reduced expression of *PKC*β1 is associated with ageing (Carbone and Tatone, 2009)	3.71 ± 0.04^*^
*PKCZ*	Protein Kinase C, Zeta	Involved in the insulin pathway; decreased expression in PCOS patients (Diamanti-Kandarakis, 2008; Rivero et al., 2012)	−1.61 ± 0.06
*PKD3*	Protein kinase, D3	Implicated in organogenesis (Ellwanger et al., 2008)	5.11 ± 0.04^*^
*RELA*	v-rel reticuloendotheliosis viral oncogene homolog A	Downregulated in women with preeclampsia (Hansson et al., 2006). Upregulated in ovarian carcinoma, leading to overexpression of COX-2 (Niesporek et al., 2007)	3.72 ± 0.04^*^
*RRAS2*	Related RAS viral oncogene homolog 2	Oncogene, defects in RRAS2 increase susceptibility to ovarian cancer (Chan et al., 1994; Fan and Richards, 2010)	7.45 ± 0.07^*^
*TLR4*	Toll-like receptor 4	Expressed by granulosa cells, mediating the effect of bacterial infection on impaired ovarian follicle growth and function (Herath et al., 2007)	2.35 ± 0.1^*^

*Fold changes are reported as mean ± SEM*.

* p < 0.05.

### Protein analysis by western blotting

**Figure 3:**
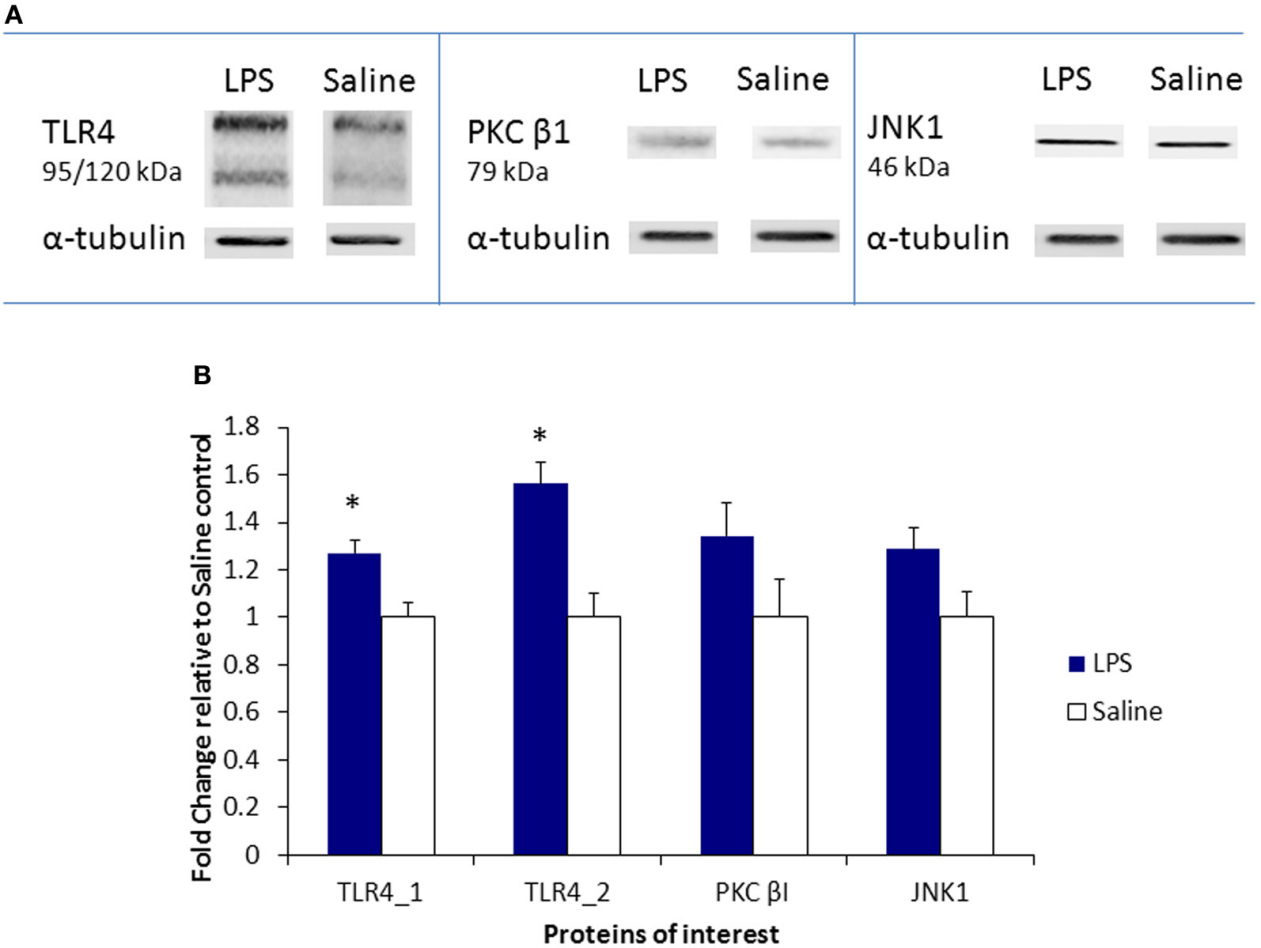
**Protein expression of TLR4, PKCβ, and JNK1 in the neonatal ovary. (A)** Changes in protein expression, as identified by immunoblotting analysis, are presented as a fold change relative to the saline control. **(B)** Representative immunoblots demonstrate the effect of LPS and saline treatments. Filled bars represent LPS-treated rats, hollow bars represent saline controls. Values are mean ± SEM. ^*^p < 0.05.

To further validate the microarray results, immunoblotting analysis was performed to examine protein expression of three significantly upregulated genes (*TLR4*; *PKC*β; MAPK8/JNK1) in the LPS-stimulated MAPK signaling pathway. Densitometry analysis of protein expression in the ovaries of LPS-treated rats, as compared to α-tubulin, revealed a significant increase of two protein bands associated with TLR4 expression [*F*_(1, 5)_ = 10.2; *F*_(1, 5)_ = 17.06, *p* < 0.05 for both]. No significant differences were evident in the expression of PKCβ1, however, a trend was observed in increased expression of JNK1 protein in the ovaries of LPS-treated animals [*F*_(1, 5)_ = 4.12, *p* = 0.09; see Figure 3B]. Representative immunoblots are presented in Figure 3A.

### Immunohistochemical analysis

**Figure 4:**
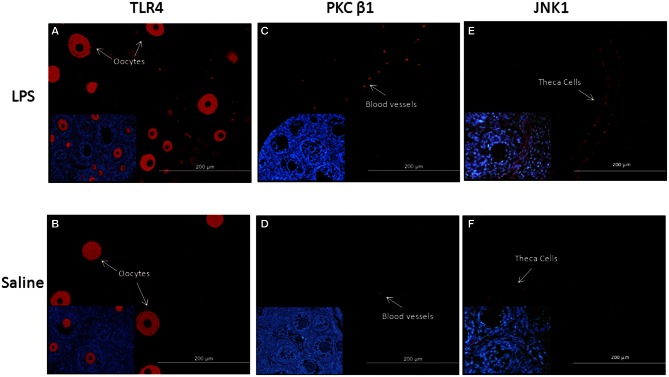
**Fluorescent immunolocalization of TLR4, PKCβ, and JNK1 protein in the ovaries of LPS and saline-treated animals. (A–B)** Represent TLR4 immunolabeling detected in the oocytes; **(C–D)** Represent PKCβ1 immunolabeling in ovarian blood vessels; **(E–F)** Represent JNK1 immunolabeling in theca cells. Insert at higher magnification is located at the bottom left corner of each representative micrograph. Blue staining (DAPI) represents nuclear staining; red staining represents specific staining for the protein of interest.

To detect localization of TLR4, PKCβ1 JNK1, in the neonatal ovary, observational immunohistochemical staining was performed in the ovaries of LPS and saline-treated animals, obtained on PND14. As demonstrated in Figures 4A,B, TLR4 immunolabeling was detected in the oocytes. In the ovaries of LPS-treated animals TLR4 was also detected in ovarian blood vessels, suggesting activation of local immune cells. PKCβ1 staining was evident in the ovarian blood vessels, to a greater extent in the ovaries of LPS-treated animals (Figures 4C,D). JNK1 immunolabeling was detected in theca cells, in both treated and control samples (Figures 4E,F).

LPS and saline-treated ovaries were also probed for markers of follicular activation and cell death. PCNA staining was equally detected in the granulosa cells and the oocytes of primordial, primary and secondary follicles in both treatment and saline control group. TUNEL staining did not reveal any significant apoptosis in either of the experimental groups (data not shown).

## Discussion

In this study we assessed, for the first time, the effect of immune challenge by peripheral administration of LPS on the ovarian transcriptome in neonatal rats. Microarray analysis revealed specific activation of immune-related pathways, such as virus entry via endocytosis, NF-κB activation by viruses, role of pattern recognition receptors in recognition of bacteria and viruses, LPS-stimulated MAPK pathway and MSP-RON signaling pathway. Exposure to LPS upregulated expression of genes implicated in cell signaling, immune cell trafficking, inflammatory response as well as reproductive development and disease, suggesting dysregulation of inflammatory processes in response to peripheral LPS exposure may occur also locally in the ovary, contributing directly to programming of reproductive health.

Given the specific relevance of LPS-stimulated MAPK pathway to the model of neonatal LPS exposure, microarray results were confirmed and elaborated on the altered components of this pathway. Activation of MAPK signaling by LPS binding of TLR4 results in transcriptional regulation and synthesis of inflammatory agents, such as tumor necrosis factor-α (TNF-α), interleukin 1β (IL-1β) and IL-6, as well as nitric oxide synthase-2 (NOS2) and cyclooxygenase-2 (COX2) (Nagano et al., 2002; Kawai and Akira, 2007). Other downstream targets include phosphoinositide-3-kinase (PI3K), which stimulation enhances activity of protein kinase C (PKC) (Frey et al., 2006). In the current study, several subunits of these genes, identified with the MAPK pathway, were upregulated in the ovaries of LPS-treated animals.

PI3K has been reported to induce activation of the serine/threonine kinase Akt, also known as protein kinase B (PKB), which in turn stimulates the mammalian target of rapamycin (mTOR) protein. Overactivation of PI3K/Akt/mTOR signaling pathway, induced by exposure to carcinogens and xenobiotics, has been implicated in premature activation of primordial follicles, leading to early follicular atresia (McLaughlin and Sobinoff, 2010; Sobinoff et al., 2010, 2011). Activation of PI3K/Akt has also been associated with the development of PCOS (Lima et al., 2006). While in the current study immunohistological assessment for markers of follicular activation and atresia revealed no significant alterations were induced by neonatal LPS exposure, our previous findings have indicated diminished population of primordial follicles in the late neonatal period in the ovaries of LPS-treated females (Sominsky et al., 2012a). These data suggest that apoptotic processes may occur early on, during the acute response to the LPS immune challenge, hindering follicular development.

The current model of neonatal LPS administration induces an acute pro-inflammatory response which involves the release of pro-inflammatory cytokines (i.e., TNF-α, IL-6, IL-1β), and this response typically resolves within 3–6 h from the time of drug administration, followed then by an anti-inflammatory release of glucocorticoids (Walker et al., 2004, 2009b). This brief activation of the immune response has been recently shown to impact on the development of the immune, reproductive, endocrine, metabolic, nervous and other physiological systems (Walker et al., 2010; Wu et al., 2011; Amath et al., 2012; Sominsky et al., 2012a, 2013). In the rat, the primordial follicle pool undergoes its final stages of formation and assembly postnatally, continuing till approximately day 3 after birth (Rajah et al., 1992; Skinner, 2005). This process is considered to be gonadotropin independent, supported and guided by growth factors and cytokines, one of which is the proinflammatory cytokine TNF-α. Previous research has demonstrated that ovarian TNF-α in the neonatal rat has an important role in determining the finite size of the primordial follicle pool. It has been demonstrated *in vitro* that TNF-α can decrease the number of oocytes and primordial follicles, via induction of apoptosis (Morrison and Marcinkiewicz, 2002). The formation of primordial follicles is then followed by the initial event of follicular development—transition to the primary follicle stage (Fortune et al., 2000; Skinner, 2005). As opposed to the later stages of follicular development, the transition from primordial to primary stage is hormone independent (Skinner, 2005). Exposure to LPS in adulthood has been also shown to induce follicular atresia *in vivo* in mice (Bromfield and Sheldon, 2013) and rats (Besnard et al., 2001) and *in vitro* in cattle (Bromfield and Sheldon, 2013). Interestingly, LPS-induced atresia is particularly detrimental to the population of primordial follicles (Bromfield and Sheldon, 2013), indicating that depletion of the primordial follicle pool during early development would subsequently impact the ovarian reserve and result in impaired fertility in later life. To our knowledge, no studies have assessed the gene expression of ovarian inflammatory pathways which were examined in the current study. While it is likely that similar changes would be evident in response to adult LPS exposure, the impact of such changes during early development, when the ovary has not yet morphologically and functionally developed, has significantly stronger implications for the growing follicle populations. It is therefore plausible to suggest that the neonatal LPS treatment instantly interferes with the delicate process of primordial follicle pool assembly and the subsequent activation of follicular development, through stimulation of an acute pro-inflammatory response. While this might be a transient perturbation, it ultimately leads to long lasting alterations in the size of the follicular pool, as observed in previous studies (Wu et al., 2011; Sominsky et al., 2012a). Even though no evidence of primordial follicle activation or apoptosis was evidenced in the current study, these processes might have occurred at an earlier time-point. Therefore, further investigation is required to clarify the time-course of apoptotic processes leading to the diminished follicular reserve reported in later life following neonatal exposure to LPS.

Activation and growth of primordial follicles are stimulated by several signaling pathways, including the MAPK and PKC (Jin et al., 2005; Du et al., 2012). The expression of several PKC isoforms (PKC α, β, δ, and ζ) was previously identified in the rat ovary (Cutler et al., 1994). In the neonatal rat ovary, MAPK, and PKC signaling cascade is involved in maturation of primordial follicles (Du et al., 2012). In the current study increased gene expression of MAPK8 and PKCβ in the ovaries of LPS-treated animals was determined by the microarray analysis and further confirmed by qRT-PCR. No significant changes in the expression of MAPK8 and PKCβ1 proteins were evident at the same time-point. However, significant increase in protein expression of TLR4 was found in the LPS-treated ovaries, and was associated with the increased mRNA levels of this gene.

TLR4, pathogen-associated molecular pattern recognition receptor, is expressed by innate immune and tissue specific cells, and recognizes bacterial molecules (e.g., LPS) (Medzhitov, 2001). While TLR4 activation is critical for generation of both innate and adaptive immune responses, its inappropriate activation can be harmful (Peroval et al., 2013). TLR4s are present in the ovary and expressed by ovarian surface epithelial cells, granulosa/cumulus cells (Herath et al., 2007; Liu et al., 2008; Richards et al., 2008), as well as by ovarian macrophages (Zhou et al., 2009), and play an essential role in regulation of fertility, through the support of ovulation and sperm capacitation (Liu et al., 2008; Shimada et al., 2008). Ovarian TLR4 signaling is normally induced by endogenous ligands, one of such is hyaluronan (HA)-rich matrix (Richards et al., 2008). Its synthesis during the ovulatory process is initiated by the surge of luteinising hormone (LH). HA-rich matrix is then recognized by cumulus cells, inducing inflammation and expression of innate immune-related genes, leading to the release of prostaglandins, TNF-α, IL-6 and other cytokines, and chemokines (Liu et al., 2008; Richards et al., 2008). These inflammatory agents activate chemokine receptors present on sperm, inducing sperm capacitation and motility (Shimada et al., 2008). Even though regulated production of cytokines is essential for ovulation and successful fertilization, dysregulation of cytokine production can impair fertility (Richards et al., 2008). In human patients, the existence of endometriosis was found to be associated with increased levels of IL-6 in circulation and in the follicular fluid, and is proposed to be related to the impaired follicular development and decreased oestradiol production, which results in infertility (Garrido et al., 2000; Pellicer et al., 2000). Activation of ovarian TLR4 by LPS has been studied in various animal and *in vitro* models. For instance, impaired antral follicle growth and function, as well as suppressed oestradiol production were reported in response to uterine *E. coli* infection, intravenous infusion with LPS and following exposure of granulosa cells to LPS *in vitro*, in cattle and sheep (Battaglia et al., 2000; Herath et al., 2007; Sheldon et al., 2009). A recent study in adult mice has demonstrated that LPS-induced atresia of primordial follicles is mediated via TLR4, since no follicle atresia was evident in *TLR4*-deficient mice (Bromfield and Sheldon, 2013).

The long-term effect of neonatal LPS exposure on TLR4 expression has been previously assessed in adult rats. Increased TLR4 mRNA expression was evident in the spleen and liver, two primary organs responsible for the synthesis of pro-inflammatory cytokines (Tracey, 2002), of neonatally-treated animals (Mouihate et al., 2010). In addition, increased expression of COX-2, the principal target of TLR4 activation, in the liver of LPS-treated animals was associated with a transient rise in circulating prostaglandin and increased activation of the HPA axis, in response to adult LPS exposure (Mouihate et al., 2010). These outcomes suggest that early life exposure to LPS has a persistent programming effect on the neuroimmune axis and this occurs via activation of TLR4. The current findings report that postnatal LPS challenge results in increased mRNA and protein expression of TLR4 in the neonatal ovary. In addition to its role in the LPS-stimulated MAPK pathway, TLR4 was associated with a number of inflammatory pathways activated in response to the neonatal treatment, as revealed by the canonical pathways analysis (i.e., role of pattern recognition receptors, MSP-RON and NF-κ B signaling pathways). Given the important regulatory role of TLR4 in reproductive function, activation of the receptor and the subsequent pro-inflammatory cascade in early life are most likely to have significant effects on ovarian development. Imbalance in the internal ovarian milieu may potentially interrupt the delicate process of follicular formation and growth, compromising reproductive capacity. Importantly, the process of primordial follicle assembly and development is highly conserved between mammals, and similar regulation of this process is considered to occur in different species, including humans (Skinner, 2005). Thus, while further studies are required to elucidate the immune mechanisms involved in the impaired ovarian development and functioning, our results importantly suggest that exposure to an immune challenge in early life, resulting in activation of TLR-related inflammatory pathways, may have significant consequences for programming of reproductive health in later life. Moreover, given that alterations in TLR4 expression are associated with pathological outcomes of common bacterial infections, such as *Escherichia coli* and *Chlamydia trachomatis*, including impaired fertility (Herath et al., 2009; Laisk et al., 2010), the current findings provide a valuable insight into the link between early life infection and fertility.

### Conflict of interest statement

The authors declare that the research was conducted in the absence of any commercial or financial relationships that could be construed as a potential conflict of interest.

## Appendix

A graphical representation of the top canonical pathways that were significantly upregulated by neonatal LPS treatment, as identified by Ingenuity Pathway Analysis. Red shading indicates up-regulation of the gene. No color indicates genes that form the pathway and have no change.
